# The Brain Tumor Segmentation (BraTS) Challenge 2023: *Focus on Pediatrics (CBTN-CONNECT-DIPGR-ASNR-MICCAI BraTS-PEDs)*

**Published:** 2024-01-04

**Authors:** Anahita Fathi Kazerooni, Nastaran Khalili, Xinyang Liu, Debanjan Haldar, Zhifan Jiang, Syed Muhammed Anwar, Jake Albrecht, Maruf Adewole, Udunna Anazodo, Hannah Anderson, Sina Bagheri, Ujjwal Baid, Timothy Bergquist, Austin J. Borja, Evan Calabrese, Verena Chung, Gian-Marco Conte, Farouk Dako, James Eddy, Ivan Ezhov, Ariana Familiar, Keyvan Farahani, Shuvanjan Haldar, Juan Eugenio Iglesias, Anastasia Janas, Elaine Johansen, Blaise V Jones, Florian Kofler, Dominic LaBella, Hollie Anne Lai, Koen Van Leemput, Hongwei Bran Li, Nazanin Maleki, Aaron S McAllister, Zeke Meier, Bjoern Menze, Ahmed W Moawad, Khanak K Nandolia, Julija Pavaine, Marie Piraud, Tina Poussaint, Sanjay P Prabhu, Zachary Reitman, Andres Rodriguez, Jeffrey D Rudie, Ibraheem Salman Shaikh, Lubdha M. Shah, Nakul Sheth, Russel Taki Shinohara, Wenxin Tu, Karthik Viswanathan, Chunhao Wang, Jeffrey B Ware, Benedikt Wiestler, Walter Wiggins, Anna Zapaishchykova, Mariam Aboian, Miriam Bornhorst, Peter de Blank, Michelle Deutsch, Maryam Fouladi, Lindsey Hoffman, Benjamin Kann, Margot Lazow, Leonie Mikael, Ali Nabavizadeh, Roger Packer, Adam Resnick, Brian Rood, Arastoo Vossough, Spyridon Bakas, Marius George Linguraru

**Affiliations:** 1 Center for Data-Driven Discovery in Biomedicine (D3b), Children’s Hospital of Philadelphia, Philadelphia, PA, USA; 2 Department of Neurosurgery, University of Pennsylvania, Philadelphia, PA, USA; 3 Center for AI & Data Science for Integrated Diagnostics (AI2D) and Center for Biomedical Image Computing and Analytics (CBICA), University of Pennsylvania, Philadelphia, PA, USA; 4 Sheikh Zayed Institute for Pediatric Surgical Innovation, Children’s National Hospital, Washington DC, USA; 5 Department of Neurosurgery, Thomas Jefferson University Hospital, PA, USA; 6 Sage Bionetworks, USA; 7 Medical Artificial Intelligence (MAI) Lab, Crestview Radiology, Lagos, Nigeria; 8 Montreal Neurological Institute (MNI), McGill University, Montreal, QC, Canada; 9 Department of Radiology, Perelman School of Medicine, University of Pennsylvania, Philadelphia, PA, USA; 10 Department of Pathology and Laboratory Medicine, Perelman School of Medicine, University of Pennsylvania, Philadelphia, PA, USA; 11 Department of Neurosurgery at the University of Southern California, CA, USA; 12 Department of Radiology, Duke University Medical Center, USA; 13 Mayo Clinic, MN, USA; 14 Center for Global Health, Perelman School of Medicine, University of Pennsylvania, Philadelphia, PA, USA; 15 Department of Informatics, Technical University Munich, Germany; 16 TranslaTUM - Central Institute for Translational Cancer Research, Technical University of Munich, Germany; 17 Cancer Imaging Program, National Cancer Institute, National Institutes of Health, Bethesda, MD, USA; 18 Biomedical Engineering Rutgers University, New Brunswick, NJ, USA; 19 Athinoula A Martinos Center for Biomedical Imaging, Massachusetts General Hospital, Boston, MA, USA; 20 Yale University, New Haven, CT, USA; 21 PrecisionFDA, U.S. Food and Drug Administration, Silver Spring, MD, USA; 22 Cincinnati Children’s Hospital Medical Center; 23 Helmholtz AI, Helmholtz Munich, Germany; 24 Department of Radiation Oncology, Duke University Medical Center, USA; 25 Department of Radiology, Children’s Health Orange County, CA, USA; 26 Department of Applied Mathematics and Computer Science, Technical University of Denmark, Denmark; 27 Department of Radiology, Nationwide Children’s Hospital, OH,; 28 Booz Allen Hamilton, McLean, VA, USA; 29 Biomedical Image Analysis & Machine Learning, Department of Quantitative Biomedicine, University of Zurich, Switzerland; 30 Department of Neuroradiology, Technical University of Munich, Munich, Germany; 31 Mercy Catholic Medical Center, Darby, PA, USA; 32 Department of Diagnostic and Interventional Radiology, All India Institute of Medical Sciences, Rishikesh, India; 33 Department of Paediatric Radiology, Royal Manchester Children’s Hospital, Manchester University Hospitals NHS Foundation Trust, University of Manchester, Manchester, UK; 34 Division of Informatics, Imaging & Data Sciences, School of Health Sciences, Faculty of Biology, Medicine and Health, University of Manchester, Manchester Academic Health Science Centre, Manchester, UK; 35 Dana-Farber Brigham Cancer Center & Boston Children’s Hospital, Boston, MA, USA; 36 Division of Neuroradiology, Department of Radiology, Boston Children’s Hospital, Harvard Medical School, Boston, Massachusetts, USA; 37 Department of Diagnostic & Interventional Imaging, Neuroradiology section. The University of Texas Health Sciences Center at Houston.; 38 University of California, San Diego, CA, USA; 39 Beth Israel Deaconess Medical Center, Harvard Medical School, MA, USA; 40 University of Utah, UT, USA; 41 Department of Radiology, The Children’s Hospital of Philadelphia, PA, USA; 42 Center for Clinical Epidemiology and Biostatistics, University of Pennsylvania, Philadelphia, USA; 43 College of Arts and Sciences, University of Pennsylvania, PA, USA; 44 Brain Tumor Institute, Children’s National Hospital, Washington, DC, USA; 45 Brain Tumor Center, Cincinnati Children’s Hospital, Cincinnati, OH, USA; 46 Pediatric Neuro-Oncology Program, Nationwide Children’s Hospital, Columbus, OH, US; 47 Center for Cancer and Blood Disorders, Phoenix Children’s Hospital, Phoenix, AZ, US; 48 Center for Cancer and Blood Disorders, Children’s National Hospital, Washington, DC, USA; 49 Departments of Radiology and Pediatrics, George Washington University School of Medicine and Health Sciences, Washington, DC, USA

**Keywords:** BraTS, BraTS-PEDs, challenge, pediatric, brain, tumor, segmentation, volume, deep learning, machine learning, artificial intelligence, AI

## Abstract

Pediatric tumors of the central nervous system are the most common cause of cancer-related death in children. The five-year survival rate for high-grade gliomas in children is less than 20%. Due to their rarity, the diagnosis of these entities is often delayed, their treatment is mainly based on historic treatment concepts, and clinical trials require multi-institutional collaborations. The MICCAI Brain Tumor Segmentation (BraTS) Challenge is a landmark community benchmark event with a successful history of 12 years of resource creation for the segmentation and analysis of adult glioma. Here we present the CBTN-CONNECT-DIPGR-ASNR-MICCAI BraTS-PEDs 2023 challenge, which represents the first BraTS challenge focused on pediatric brain tumors with data acquired across multiple international consortia dedicated to pediatric neuro-oncology and clinical trials. The BraTS-PEDs 2023 challenge focuses on benchmarking the development of volumentric segmentation algorithms for pediatric brain glioma through standardized quantitative performance evaluation metrics utilized across the BraTS 2023 cluster of challenges. Models gaining knowledge from the BraTS-PEDs multi-parametric structural MRI (mpMRI) training data will be evaluated on separate validation and unseen test mpMRI dataof high-grade pediatric glioma. The CBTN-CONNECT-DIPGR-ASNR-MICCAI BraTS-PEDs 2023 challenge brings together clinicians and AI/imaging scientists to lead to faster development of automated segmentation techniques that could benefit clinical trials, and ultimately the care of children with brain tumors.

## 1. Introduction

Although rare, pediatric tumors of thecentral nervous system are the most common cause of cancer-related death in children. While pediatric brain tumors may share certain similarities with adult brain tumors, their imaging and clinical presentations differ. For example, adult glioblastomas (GBMs), pediatric diffuse midline gliomas (DMGs, including pediatric diffuse intrinsic pontine glioma (DIPGs)) are high grade gliomas with short average overall survival [1,2]. The incidence of GBMs is 3 in 100,000 people, while DMGs are about three times more rare. While GBMs are usually found in the frontal or/and temporal lobes at an average age of 64 years, many DMGs are located in the pons and often diagnosed between 5 and 10 years of age. Enhancing tumor regions on post-gadolinium T1-weighted MRI and radiologically-defined necrotic tissue regions are common imaging findings in GBM, but less common or clear in DMGs, at least at initial diagnosis. Thus, pediatric brain tumors require dedicated imaging tools that help in their characterization and facilitate their diagnosis/prognosis [3–5].

Tumor segmentation is essential in surgical and treatment planning, as well as in response assessment and longitudinal monitoring. However, manual segmentation is time-consuming and has high inter-operator variability [6]. Pediatric brain tumors are variable in their aggressiveness, prognosis, and heterogeneous histologic subregions, i.e., peritumoral edematous/invaded tissue, necrotic core, and enhancing tumor core, as reflected in their radio-phenotypes on multi-parametric magnetic resonance imaging (mpMRI) scans. Some pediatric brain tumors, such as DIPGs, are heterogeneous and typically located in surgically inaccessible anatomical locations, leaving them unresectable. As such, assessment of tumor progression is mainly based on the changes in size of the tumor measured from longitudinal scans. The current standard, as recommended by the Response Assessment in Pediatric Neuro-Oncology (RAPNO) cooperative working group [7,8], is based on two-dimensional (2D) linear measurements of the tumor subregions (in the axial slice of the largest tumor extent), including enhancing tumor, non-enhancing tumor, cystic components, and peritumoral edematous tissue. Nonetheless, these 2D measurements are inaccurate surrogates of the volume and they cannot accurately account for irregularity of the tumor shape and appearance and hence further increase the inter-operator variability. Studies in adult brain tumors have indicated superiority of 3D volumetric measurement of tumor extent in the prediction of clinical endpoints over 2D methods [9]. While the importance of volumetric tumor measurements, as compared to 2D ones, are being increasingly acknowledged in response assessment of pediatric brain tumors [2,10], there is a lack of available automated tools to facilitate the cumbersome task of segmentation of tumor subregions on assocaited scans. There are only a handful of automated tumor segmentation methods explicitly proposed for pediatric brain tumors [3,11–17]. However, the majority of these methods have been developed only for the segmentation of the T2 fluid attenuated inversion recovery (FLAIR) abnormal signal [11,12,17], also called whole tumor (WT) in the BraTS terminology. Furthermore, none of the proposed methods has been evaluated and compared on the same validation data and through a controlled study design, and therefore there is a gap in benchmarking the automated segmentation tools in pediatric brain tumors.

The MICCAI brain tumor segmentation (BraTS) challenges have established a community benchmark dataset and environment for adult glioma over the past 11 years [18–21]. The focus of this year’s BraTS is expanded to a Cluster of Challenges spanning across various tumor entities, missing data, and technical considerations. During BraTS 2022, we organized the first initiative to include pediatric brain tumors, specifically DMGs, in the test phase of the BraTS 2022 challenge. The findings of this evaluation study encouraged us to organize a larger and more diverse initiative in 2023 with multi-institutional pediatric data. In the BraTS-PEDs 2023 challenge, the focus is on pediatric brain tumor entities. We have created a retrospective multi-institutional (multi-consortium) pediatric database, with the data collected through a few consortia, including Children’s Brain Tumor Network (CBTN, https://cbtn.org/) [22], DIPG Registry (https://www.dipgregistry.org) and the COllaborative Network for NEuro-oncology Clinical Trials (CONNECT, https://connectconsortium.org/). Additional data from participating pediatric institutions have been included in the BraTS-PEDs cohort. The American Society of Neuroradiology (ASNR, https://www.asnr.org/) collaborated in generating ground truth annotation for the majority of data in this challenge. These data will be collectively used to develop and quantitatively evaluate algorithms developed and models trained by the challenge participants in unseen validation data until July 2023, when the organizers will stop accepting new submissions and evaluate submitted containerized algorithms in a completely unseen pediatric patient population. This manuscript provides details about the CBTN-CONNECT-ASNR-MICCAI BraTS-PEDs 2023 challenge, as the first benchmarking initiative on pediatric brain tumor segmentation.

## 2. Materials & Methods

### 2.1 Data

The BraTS-PEDs 2023 dataset includes a retrospective multi-institutional cohort of conventional/structural magnetic resonance imaging (MRI) sequences, including pre- and post-gadolinium T1-weighted (labeled as T1 and T1CE), T2-weighted (T2), and T2-weighted fluid attenuated inversion recovery (T2-FLAIR) images, from 228 pediatric high-grade glioma. These conventional multiparametric MRI (mpMRI) sequences are commonly acquired as part of standard clinical imaging for brain tumors. However, the image acquisition protocols and MRI equipment differ across different institutions, resulting in heterogeneity in image quality in the provided cohort. Inclusion criteria comprised of pediatric subjects with: (1) histologically-approved high-grade glioma, i.e., high-grade astrocytoma and diffuse midline glioma (DMG), including radiologically or histologically-proven diffuse intrinsic pontine glioma (DIPG); (2) availability of all four structural mpMRI sequences on treatment-naive imaging sessions. Exclusion criteria consisted of: (1) images assessed to be of low quality or with artifacts that would not allow for reliable tumor segmentation; and (2) infants younger than one month of age. Data for n = 228 patients was obtained through CBTN (n = 138), Boston’s Children Hospital (n = 61), and Yale University (n = 29).

The cohort included in the BraTS-PEDs 2023 challenge is split into training, validation, and testing datasets. The data shared with the participants comprise mpMRI scans and ground truth labels for the training cohort, as well as mpMRI sequences without any associated ground truth for the validation cohort. Notably, the testing data that will be used for evaluating the performance of the methods submitted by challenge participants will not be shared with the participants, but the containerized submissions of the participants will be evaluated by the synapse.org platform, powered by MedPerf [23].

Participants are prohibited from training their algorithm on any additional public and/or private data (from their own institutions) besides the provided BraTS-PEDs 2023 data, or using models pretrained on any other dataset. This restriction is imposed to allow for a fair comparison among the participating methods. However, participants can use additional public and/or private data only for publication of their scientific papers, if they also provide a report of their results on the data from BraTS-PEDs 2023 challenge alone, and discuss potential differences in the obtained results.

#### 2.1.1 Imaging Data Description

For all patients, mpMRI scans were pre-processed using a standardized approach, including conversion of the DICOM files to the NIfTI file format [24], co-registration to the same anatomical template (SRI24) [25], and resampling to an isotropic resolution (1*mm*^3^). The pre-processing pipeline is publicly available through the Cancer Imaging Phenomics Toolkit (CaPTk) ^1^ [26–28] and Federated Tumor Segmentation (FeTS) tool ^2^. De-identification was performed through removing protected Health Information (PHI) from DICOM headers in DICOM to NIfTI conversion step [29, 30]. De-facing was performed via skull-stripping using a formerly proposed pediatric-specific skull-stripping method [3] to prevent any potential facial reconstruction/recognition of the patients.

**Fig.1:**
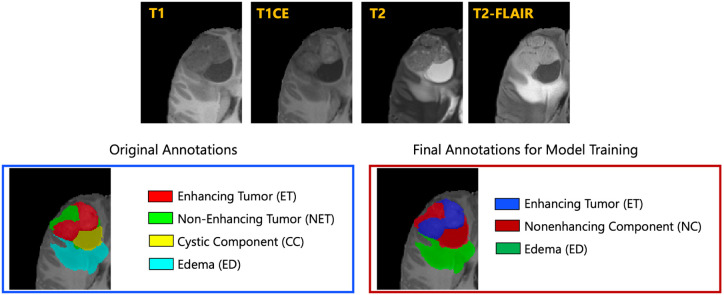
Illustrative example of tumor subregions in pediatric brain tumors. Image panels with the annotated tumor subregions along with mpMRI structural scans (T1, T1CE, T2, and T2-FLAIR). The left-most side image on the bottom panel with the overlaid annotations showcases the original tumor subregions, i.e., enhancing tumor (ET - red), non-enhancing tumor (NET - green), cystic component (CC - yellow), and edema (ED - teal). The image on the right hand side of the bottom panel with the overlaid annotations on the T2 sequence demonstrates the tumor subregions provided to the BraTS-PEDs 2023 participants: enhancing tumor (ET), nonenhancing component (NC), and edema (ED) [3].

The resulted images were segmented into tumor subregions using an integration of two pediatric automated deep learning segmentation models [3,31]. The result of this step was segmentation of the tumors into four main subregions, recommended by RAPNO working group for evaluation of the treatment response in high-grade gliomas and DIPGs. The annotated tumor subregions comprised of enhancing tumor (ET), nonenhancing tumor (NET), cystic component (CC), and peritumoral edema (ED) regions. ET is described by areas with enhancement (brightness) on T1 post-contrast images as compared to T1 pre-contrast. In case of mild enhancement, checking the signal intensity of normal brain structure can be helpful. CC typically appears with hyperintense signal (very bright) on T2 and hypointense signal (dark) on T1CE. The cystic portion should be within the tumor, either centrally or peripherally (as compared to ED which is peritumoral). The brightness of CC is here defined as comparable or close to cerebrospinal fluid (CSF). NET is defined as any other abnormal signal intensity within the tumorous region that cannot be defined as enhancing or cystic. For example, the abnormal signal intensity on T1, T2-FLAIR, and T2 that is not enhancing on T1CE should be considered as nonenhancing portion. ED is defined by the abnormal hyperintense signal (very bright) on FLAIR scans. ED is finger-like spreading that preserves underlying brain structure and surrounds the tumor. The automatically-generated four labels (Figure 1) using the automated segmentation tool were used as preliminary segmentation to be manually revised by volunteer neuroradiology experts of varying rank and experience, in accordance with annotation guidelines. The volunteer neuroradiology expert annotators were provided with four mpMRI sequences (T1, T1CE, T2, FLAIR) along with the fused automated segmentation volume to initiate the manual refinements. The ITK-SNAP [32] software was used for making these refinements. Once the automated segmentations were refined by the annotators, three attending board-certified neuroradiologists, reviewed the segmentations. Depending upon correctness, these segmentations were either approved or returned to the individual annotator for further refinements. This process was followed iteratively until the approvers found the refined tumor subregion segmentations acceptable for public release and the challenge conduction.

While four tumor subregions were annotated, in keeping with the other BraTS 2023 challenges and to allow the participants to incorporate data from adult glioma challenge for their model training, the participants are provided with three segmentation labels, including enhancing tumor (ET), nonenhancing component (NC - a combination of nonenhancing tumor, cystic component, and necrosis), as well as peritumoral edematous area (ED) (Figure 1).

#### 2.1.2 Common errors of automated segmentations

In our experience with automated segmentation of pediatric brain tumors, some errors may be noticed:

1. Peri-ventricular edema segmented as edema
2. Remote areas segmented as tumor (far from actual tumor region)
3. Under-segmentation of cysts
4. Non-enhancing tumor segmented as cyst, or vice versa

### 2.2 Model Training Using Generally Nuanced Deep Learning Framework (GaNDLF)

We will offer to the participants a baseline approach implemented in a modular open-source framework, namely the Generally Nuanced Deep Learning Framework (GaNDLF) [33], which is maintained by the MLCommons organization (github.com/mlcommons/gandlf). GaNDLF offers some network architectures, but also allows the user to leverage the functionality of other libraries, such as PILLOW and MONAI. The participants can decide to use GaNDLF to develop their approach or use their own custom source code. For their submission, they need to package their submitting approach in an MLCube container, instructions for which will be clearly provided in the Synapse platform. MLCube containers are automatically generated by GaNDLF and will be used to evaluate all submissions through the MedPerf platform [23] on each contributing site’s data.

### 2.3 Performance Evaluation

As described earlier and exemplified in Figure 2 (bottom panel), for the BraTS-PEDs 2023 challenge, the regions to evaluate the performances are: i) the “enhancing tumor” (ET), ii) the “enhancing tumor/cystic component/necrosis” (NC) and iii) the entire tumorous region, or the so-called “whole tumor” (WT).

The participants are required to send the output of their methods to the evaluation platform for the scoring to occur during the training and the validation phases. At the end of the validation phase the participants are asked to identify the method they would like to evaluate in the final testing/ranking phase. The organizers will then confirm receiving the containerized method and will evaluate it on withheld testing data. The participants will be provided guidelines on the form of the container as we have done in previous years. This will enable confirmation of reproducibility, comparing these algorithms to the previous BraTS instances and comparison with results obtained by algorithms of previous years, thereby maximizing solutions in solving the problem of brain tumor segmentation.

During the training and validation phases, the participants will be able to test the functionality of their submission through three platforms:

1. Cancer Imaging Phenomics Toolkit (CaPTk [5–6], https://github.com/CBICA/CaPTk)
2. Federated Tumor Segmentation (FeTS) Tool [7] (https://fets-ai.github.io/Front-End/)
3. Online evaluation platform (Synapse)

### 2.4 Participation Timeline

The challenge is composed of three main stages:

1. Training: The four MRI sequences along with the corresponding ground truth labels will be shared with participants to design and train their methods.
2. Validation: The validation data will be released to the participants within three weeks after the training data. The participants will not be provided with the ground truth of the validation data, but will be given the opportunity to submit multiple times to the online evaluation platforms. The participants can generate preliminary results for their trained models in unseen data and report them in their submitted short MICCAI LNCS papers, in addition to their cross-validated results on the training data, to be published in conjunction with the proceedings of the BrainLes workshop. The top-ranked participating teams in the validation phase will be invited to prepare their slides for a short oral presentation of their method during the BraTS challenge at MICCAI 2023.
3. Testing/Ranking: After the participants upload their containerized method in the evaluation platforms, they will be evaluated and ranked on unseen testing data, which will not be made available. The final top-ranked participating teams will be announced at the 2023 MICCAI Annual Meeting.

## 3 Discussion

In this paper, we outlined the design of the first pediatric brain tumor segmentation (BraTS-PEDs 2023) challenge, to benchmark methods devised for segmentation of pediatric brain tumors. In 2022, for the first time, the BraTS (continuous evaluation) challenge included testing the top-ranked methods, designed for segmentation of adult gliomas, on unseen held out data of pediatric brain tumors. The results from last year’s BraTS 2022 showed that while training and optimizing the models on adult brain tumors could result in relatively high Dice scores in segmentation of the whole tumor region on mpMRI scans of pediatric brain tumors, they perform poorly on segmentation of tumor subregions (e.g. in segmentation of enhancing tumor/cystic components as a counterpart to tumor core in adult brain tumors). These results supported our hypothesis that the techniques to be applied to pediatric population need to be trained (at least partly) on curated and annotated cohort of pediatric brain tumors. However, similar to any other machine and deep learning problems, training a generalizable model mandates ample dataset processed in a standardized approach. To achieve this goal, BraTS-PEDs dataset provides the largest annotated cohort of pediatric high-grade glioma (astrocytoma, and DMG/DIPGs). We are actively working on increasing the number of subjects in this cohort to provide the community with a large dataset of these rare tumors, and to facilitate the future development of tools for computer-aided treatment planning. Future BraTS-PEDs challenges will include data from more institutions and will be extended to post-operative or post-treatment scans.

## Footnotes

1
https://cbica.github.io/CaPTk/

2
https://github.com/FETS-AI/Front-End/
